# The Detection of Surfactant Proteins A, B, C and D in the Human Brain and Their Regulation in Cerebral Infarction, Autoimmune Conditions and Infections of the CNS

**DOI:** 10.1371/journal.pone.0074412

**Published:** 2013-09-30

**Authors:** Stefan Schob, Martin Schicht, Saadettin Sel, Dankwart Stiller, Alexander Kekulé, Friedrich Paulsen, Erik Maronde, Lars Bräuer

**Affiliations:** 1 Institute of Anatomy and Cell Biology, Martin Luther University Halle-Wittenberg, Halle (Saale), Germany; 2 Institute of Anatomy, Department II, Friedrich Alexander University Erlangen-Nürnberg, Erlangen, Germany; 3 Institute of Anatomy, Department III, Johann Wolfgang Goethe University, Frankfurt, Germany; 4 Department of Ophthalmology, University Heidelberg, Heidelberg, Germany; 5 Institute of Forensic Medicine, Martin Luther University Halle-Wittenberg, Halle (Saale), Germany; 6 Institute for Medical Microbiology, Martin Luther University Halle-Wittenberg, Halle (Saale), Germany; Glasgow University, United Kingdom

## Abstract

Surfactant proteins (SP) have been studied intensively in the respiratory system. Surfactant protein A and surfactant protein D are proteins belonging to the family of collectins each playing a major role in the innate immune system. The ability of surfactant protein A and surfactant protein D to bind various pathogens and facilitate their elimination has been described in a vast number of studies. Surfactant proteins are very important in modulating the host's inflammatory response and participate in the clearance of apoptotic cells. Surfactant protein B and surfactant protein C are proteins responsible for lowering the surface tension in the lungs. The aim of this study was an investigation of expression of surfactant proteins in the central nervous system to assess their specific distribution patterns. The second aim was to quantify surfactant proteins in cerebrospinal fluid of healthy subjects compared to patients suffering from different neuropathologies. The expression of mRNA for the surfactant proteins was analyzed with RT-PCR done with samples from different parts of the human brain. The production of the surfactant proteins in the brain was verified using immunohistochemistry and Western blot. The concentrations of the surfactant proteins in cerebrospinal fluid from healthy subjects and patients suffering from neuropathologic conditions were quantified using ELISA. Our results revealed that surfactant proteins are present in the central nervous system and that the concentrations of one or more surfactant proteins in healthy subjects differed significantly from those of patients affected by central autoimmune processes, CNS infections or cerebral infarction. Based on the localization of the surfactant proteins in the brain, their different levels in normal versus pathologic samples of cerebrospinal fluid and their well-known functions in the lungs, it appears that the surfactant proteins may play roles in host defense of the brain, facilitation of cerebrospinal fluid secretion and maintenance of the latter's rheological properties.

## Introduction

The healthy central nervous system has conventionally been considered to be almost completely independent of the immune system [1]. Interactions of the two systems were assumed to occur only under pathological conditions [2]. In recent years this absolute ‘immune privilege’ of the CNS has been reconsidered and a distinct network of brain specific defense- and regulatory mechanisms has been unveiled [3], [4].

In general, the blood-brain barrier (BBB) and the blood-cerebrospinal fluid barrier (BCSFB) limit the access of cells and proteins circulating in the bloodstream to the brain [5]. The restricted entry of peripherally produced cells and molecules necessitates an efficient system in the brain capable of recognizing and eliminating pathogens, apoptotic cells and detrimental metabolic products. Astrocytes and microglia are both cellular players that monitor the brain continuously and react rapidly in case of pathological changes [6], [7]. Besides these brain-specific effector cells, many molecular factors in the innate immune system, e.g. complement factors [8] and defensins [9], have been shown to be important components of the CNS.

Surfactant proteins are part of the pulmonary surfactant, a thin layer covering the alveolar surface, decreasing the surface tension between the air and the alveoli of the lungs to prevent the collapse of the small airways at the end of expiration. The pulmonary surfactant consists of lipids, mainly phosphatidylcholine and phosphatidylglycerol, and surfactant proteins A, B, C and D. The proteins are essential for the proper function of the surfactant and can be divided into two major structural and as a consequence functional groups.

Surfactant protein A (SP-A) and surfactant protein D (SP-D) are molecular factors of the innate immune system; they facilitate the elimination of numerous pathogens in the lungs. For example, they promote phagocytosis of *Mycobacterium tuberculosis* by alveolar macrophages and modulate the intracellular mechanisms of killing the invading pathogen [10]. While SP-A is upregulated in human alveolar epithelial cells after LPS exposure [11], SP-D mediates phagocytosis of *Pseudomonas aeruginosa*, *Haemophilus influenzae* and *Klebsiella pneumoniae* [12], [13], [14]. SP-A and SP-D are also part of the first line of defense against viral and fungal invaders [15], [16]. Recent studies also showed their involvement in recognition and clearance of apoptotic cells [17].

Surfactant protein B (SP-B) and surfactant protein C (SP-C) are small hydrophobic proteins with the main function of decreasing surface tension at the air-liquid interface in distal airways [18]. They interact with phospholipids (mainly dipalmitoylphosphatidylcholine) and enable the adsorption of lipids and their distribution at the air-liquid interface [19]. Furthermore, they are able to render phospholipid membranes highly permeable to polar molecules and are assumed to form proteolipid pores in biomembranes [20]. SP-B also has anti-inflammatory properties: Ikegami et al. showed that SP-B deficiency leads to an increase of proinflammatory cells, total protein and proinflammatory cytokines in the bronchioalveolar fluid of transgenic mice. Restoration of SP-B expression rapidly reversed the described signs of inflammation [21]. Besides its anti-inflammatory abilities, SP-B has bactericidal (*Staphylococcus aureus*, *Klebsiella pneumoniae*) and bacteriostatic (*Pseudomonas aeruginosa*) effects [22].

SP-C binds bacterial lipopolysaccharide (LPS) and modifies pro-inflammatory cytokine-mRNA in monocytes after LPS stimulation [23], [24].

Extra-pulmonary production of SPs and their importance for host defense and maintenance of physiological conditions has been demonstrated in a number of tissues and organs amongst them the salivary system [25], [26], the ocular surface [27], [28] as well as the nasal cavity [29].

Considering the specific localization of SPs at anatomical–physiological barriers (e.g., the air-tissue interface in the airways) and their importance as innate defense molecules, we hypothesized that the four known surfactant proteins are molecular components of the innate immune system of the CNS. We particularly assumed their role as pathogen-binding proteins at the barriers between blood and CNS, protecting the highly vulnerable tissue from bacteria, viruses and fungi circulating in the bloodstream.

In addition, regarding the known effects of SPs on biological fluids, we proposed the presence of SPs in cerebrospinal fluid, acting as regulators of its rheological properties.

## Materials and Methods

The study was conducted in compliance with Institutional Review Board regulations, informed consent regulations, and the provisions of the Declaration of Helsinki.

### Tissue

Because of the origin of tissue samples and the background of acquisition of csf samples no ethics committee approval was necessary.

The brain samples were obtained from body donors (3 female, 4 male, aged 54–77 years) at the Institute for Anatomy and Cell Biology Halle-Wittenberg, Germany. Prior to death every body donor dedicated his body to the Institute for Anatomy and Cell Biology for scientific and academic purposes, stating this in his last will. Tissue samples were anonymized. All tissues used were dissected from the cadavers within 9–24 hours post mortem. Prior to dissection, the medical history of each body donor was studied and donors with neurological syndromes were excluded. The brain of each cadaver was sagitally split in half. One half was fixed in 4% paraformaldehyde, the other half was further dissected and the regions of interests (cortex, brainstem, choroid plexus, meninges, cerebellum, optic nerve, pineal gland, pituitary gland) were immediately snap-frozen in liquid nitrogen and stored at −80°C for further biomolecular investigation.

### Cerebrospinal fluid (CSF)

Samples of cerebrospinal fluid (n = 118; 50 female, 68 male) were generously donated by the Institute of Medical Microbiology, Martin-Luther-University, Halle/Wittenberg. The samples were collected for routine medical diagnostics to confirm or disprove CNS-infections and the remnants were stored over years at −80°C for possible further testing. Informed consent was written for the lumbar puncture and the acquisition of cerebrospinal fluid. After the death of the patient the related CSF sample was approved for scientific purposes and released to the Institute for Anatomy and Cell Biology together with the information about gender and diagnoses of the patients. The CSF samples were anonymized. Samples of cerebrospinal fluid from patients with stroke (ischemic or hemorrhagic infarction of the brain), autoimmune inflammation of the CNS (multiple sclerosis, acute disseminating encephalomyelitis, neuromyelitisoptica) and infections of the CNS were included in the study. In order to sustain comprehensibility of the underlying cause for the alteration of SP concentrations in the CSF, the following specimens were excluded: CSF samples with blood contamination, CSF samples from patients with stroke/cerebral infarction plus evidence of bacterial growth, CSF samples from patients with hemorrhage into the subarachnoid space and CSF samples from patients without a diagnosed CNS illness but with a laboratory ascertained elevated CSF/serum albumin ratio, indicating a disturbance of the blood-cerebrospinal fluid barrier.

### Preparation of RNA and synthesis of complementary DNA (cDNA)

For reverse transcriptase polymerase chain reaction (RT-PCR), tissue samples from 5 different regions of the 7 donor brains were crushed in an agate mortar under liquid nitrogen and homogenized in 5 ml PeqGold RNA pure solution (PeqLab Biotechnologie, Erlangen, Germany) with a Polytron homogenizer. The CSF samples were treated under the same conditions except for crushing under liquid nitrogen. Insoluble material was removed by centrifugation (12.000 g, 5 min, 4°C). Total RNA was isolated using RNeasy-Kit (Qiagen, Hilden, Germany). Crude RNA was purified with isopropanol and repeated ethanol precipitation. Contaminating DNA was destroyed by digestion with RNase-free DNase I (20 min 25°C; Boehringer, Mannheim, Germany). The DNAse was heat-denatured for 15 min at 65°C. 500 ng RNA were used for each reaction: cDNA was generated with 50 ng/µl (20 pmol) oligo (dT)15 primer (Amersham Pharmacia Biotech, Uppsala, Sweden) and 0.8 µl superscript RNase H^−^ reverse transcriptase (100 U; Gibco, Paisley, UK) for 60 min at 37°C. The ubiquitously expressed β-actin, which proved amplifiable in each case with the specific primer pair, served as the internal control for the integrity of the transcribed cDNA.

### PCR – polymerase chain reaction

For conventional PCR, 1 µl cDNA from each sample was incubated with 13.7 µl H_2_O, 1 µl 50 mM MgCl_2_, 0.5 µl dNTPs, 2 µl 10x PCR buffer, 0.3 µl (5 U) Taq DNA polymerase (Invitrogen) and 0.5 µl (100 pmol) of each of the following primers (displayed in table 1).

**Table 1:** Sequences of the primers used for detection of surfactant proteins (A; B; C; D), RT-PCR analysis.

Primer	Sense primer, 5′ → 3′	Antisense primer, 5′ →3′	bp	°C
SP-A	CTG TCC CAA GGA ATC CAG AG	CCG TCT GAG TAG CGG AAG TC	120	57
SP-B	CAC CAT GTT CCC CAT TCC TCT	TCA TCC ATG GAG CAC CGG AGG ACG	239	57
SP-C	CTG GTT ACC ACT GCC ACC TT	TCA AGA CTG GGG ATG CTC TC	142	57
SP-D	AGG AGC AAA GGG AGA AAG TGG G	CAG CTG TGC CTC CGT AAA TGG	199	57
Gyrase A	TGT GCT TTA TGC CAT GAG CGA	TCC ACC GAA CCG AAG TTG C	220	58
β-Actin	CAA GAG ATG GCC ACG GCT GCT	TCC TTC TGC ATC CTG TCG GCA	275	60

After 5 min of heat denaturation at 96°C, the PCR cycle consisted of (1) 96°C for 60 s, (2) 57°C (SP-A, SP-B, SP-C, and SP-D) for 60 s each, (3) 72°C for 60 s. 35 cycles were performed with each primer pair. The final elongation cycle consisted of 72°C for 4 min. The primers were synthesized by MWG-Biotech AG, Ebersberg, Germany. 10 µl of the PCR product solution was loaded on an agarose gel and after electrophoresis the amplified products were visualized via fluorescence. Base pair (bp) size values were compared with gene bank data. For verification and comparison, plasmids containing the open reading frame for the corresponding protein were used as a reference (German Resource Centre for Genome Research GmbH; SP-A: IRAUp969H0686D6; SP-B: IRAKp961K1368Q2, SP-C: IKAUp969F0244D6, SP-D: IRAUp969D0386D6). PCR products were also confirmed by BigDye sequencing (Applied Biosystems, Foster City, CA). To estimate the amount of amplified PCR product, we performed a β-actin PCR with specific primers (table 1) for each investigated tissue. This additional PCR was performed under the above-mentioned conditions. 10 µl of the PCR product were loaded on the agarose gel. The base pair marker (MassRuler DNA-Ladder, Fermentas, St. Leon-Rot, Germany) used for each PCR ranges from 80 to 1,031 bp.

### Antibodies

Antibodies (displayed in table 2 and 3) were used for Western blot analysis as well as for immunohistochemical investigations as specified by the manufacturer.

**Table 2:** Molecular weights of the surfactant proteins and specific antibodies used for their detection in Western blot analysis and immunohistochemistry.

Protein	Molecular weight (kDa)	Antibody	Company, catalog number
SP-A*^1^	28–36; 66	Mouse monoclonal anti human SP-A	Millipore; MAB3270
SP-A*^2^	28–36; 66	Goat polyclonal anti human SP-A	Santa Cruz; sc-7699
SP-B*^3^	8; 18; 40	Goat polyclonal anti human SP-B	Santa Cruz; sc-7701
SP-B*^4^	8; 18; 40	Mouse monoclonal anti human SP-B	Millipore; MAB3276
SP-C*^5^	4–6; 6–12; 21; 26	Rabbit polyclonal anti human SP-C	Chemicon; AB3786
SP-D*^6^	43	Rabbit polyclonal anti human SP-D	Chemicon; AB3434
SP-D*^7^	43	Rabbit polyclonal anti human SP-D	Santa Cruz; sc-7708

**Table 3:** Specific secondary antibodies used for their detection in Western blot analysis and immunohistochemistry.

Antibody	Company, Catalog number
Anti-rabbit IgG, HRP- conjugated	Dako, Denmark; P0448
Anti-mouse IgG, HRP- conjugated	Dianova, Hamburg; 80807
Anti-rabbit IgG, biotinylated	Dako, Denmark; P0448
Anti-goat IgG, biotinylated	Dako, Denmark; P0449

### Western blot

Tissue samples (n = 4; 2 male, 2 female) of brainstem (BS), cerebellum (Ce), choroid plexus (CP), the circle of Willis (CW), subventricular cortex (Ve), pia mater (PM) from body donors were homogenized in RIPA buffer (20–188, Chemicon-Millipore) on ice with a tissue homogenizer (IKA T 10 Basic Ultra Turrax Homogenizer, Cole Parmer). Generally, 100 mg of each tissue sample was submerged in 300 µl of RIPA buffer. After homogenization the samples were incubated for 2 hours on ice and subsequently spun down at 21000 g for 20 min. The supernatant containing the proteins was used for further analysis. Two native CSF samples were used for Western blotting without prior protein isolation. The protein concentration of these samples was determined with a protein assay based on the Bradford dye-binding procedure (BioRad, Hercules, CA). For SDS-PAGE, each well of a 15% Tris-HCL gel was loaded with 40 µg of total protein. Protein isolate from human lung tissue served as positive control. After separating the proteins in the gel under reducing conditions, the proteins were transferred electrophoretically onto nitrocellulose membranes. Unspecific binding of the primary antibody was prevented using 5% milk in PBS as blocking solution for 1 hour at room temperature. The blocking solution was removed and the membranes were incubated with the antibodies for SP-A^*1^ (1∶500), SP-B^*4^ (1∶200), SP-C^*5^ (1∶500), SP-D^*6^ (1∶500) at 4°C for 12 hours (displayed in table 2 and 3). Subsequently, the membrane was washed and the corresponding secondary antibody (HRP-conjugated) was added in a final dilution of 1∶2500 for an incubation period of 1 hour at room temperature. Positive immunoreactivity was detected using chemiluminescence (ECL plus; Amersham-Pharmacia, Uppsala, Sweden) with the IVIS 100 Imaging System (Xenogen Imaging Technologies). The molecular weights of the detected protein bands were estimated using standard proteins (Prestained Protein Ladder, Fermentas, St. Leon-Rot, Germany) ranging from 11 to 170 kDa. To proof for bacterial contamination all liquor and tissue samples used were checked for presence of gyrase A using the regarding antibody [30]. We were unable to find significant amounts of bacterial contamination in any of the samples.

### Immunohistochemistry

For immunohistochemistry, tissue specimens (N = 4; 2 female, 2 male cadavers) from brainstem, cerebellum, choroid plexus, pia mater, pineal gland, pituitary gland and Subventricular cortex were embedded in paraffin, sectioned (6 µm) and deparaffinized. Immunohistochemical staining was performed with antibodies to SP-A^*2^, SP-B^*3^, SP-C^*5^ and SP-D^*7^ (displayed in table 2 and 3). Antigen retrieval was performed by microwave pretreatment for 10 min and nonspecific binding inhibited by incubation with normal serum (Dako, corresponding to the species, in which the secondary antibody was produced) 1∶5 in Tris buffered saline (TBS). Each primary antibody was applied overnight at 4°C. The secondary antibodies were incubated at room temperature for 2 h. Visualization was achieved with peroxidase-labeled streptavidin-biotin and diaminobenzidine (DAB) for at least 5 min. After counterstaining with Mayer's hematoxylin, the sections were mounted in Aquatex (Boehringer, Mannheim, Germany). Two negative control sections were used in each case: one was incubated with the secondary antibody only, and the other with the primary antibody only. Sections of human lung served as positive control. The slides were examined with a Keyence Biozero BZ8100E microscope.

### ELISA – Enzyme-linked immunosorbent assay

Commercially available enzyme-linked immunosorbent assay kits (USCN, Wuhan, China) were used to quantify the amount of SP-A (E90890Hu, ELISA Kit for Surfactant Associated Protein A), SP-B (E91622Hu, ELISA Kit for Surfactant Associated Protein B), SP-C (E91623Hu, ELISA Kit for Surfactant Associated Protein C) and SP-D (E91039Hu, ELISA Kit for surfactant-associated protein D) in samples of cerebrospinal fluid and protein extracts from human brain tissue. The analysis was performed using a microplate spectrophotometer (ELISA-reader) at a wavelength of 450 nm and 405 nm for measuring the absorbance. By comparing with the standard series and the determined values for antigen concentration (protein concentration), each sample was calculated as surfactant protein in ng/total protein in mg (ng/mg).

### Statistical analysis

After evaluating the values on normal distribution using Kolmogorov-Smirnov test we performed Mann-Whitney U test to compare the means among different groups. For the graphical representations we used box plots showing median value and 50% interquartile interval for SP-A, SP-B, SP-C, SP-D concentration in ng/mg for the different groups. The hinge values are ±1.5 standard deviation (SD) about the mean value, the data points indicated by unfilled circles are between 1.5 and 3 SD from the mean value and outliner data points (>3 SD) are shown as stars. The number next to the unfilled circle or star indicates to the case number in the raw data sheet. The PASW SPSS 18 (IBM) for windows® software package was used for the statistical analysis. A p value<0.05 was considered statistically significant.

The analyzed CSF samples were sorted into one out of four main categories I) autoimmune diseased, II) infection diseased, III) cerebral infarction, IV) healthy. The medical history of every subject related to an analyzed CSF sample was studied; thereupon the CSF sample was categorized in one of these four groups.

CSF samples were included into the ‘autoimmune diseased’ group if the sample was derived from a patient currently diagnosed with ongoing multiple sclerosis, acute disseminated encephalmomyelitis or neuromyelitisoptica.

CSF samples were included into the ‘infection diseased’ – group if the sample was derived from a patient diagnosed with current CNS infection of if the suspected diagnosis was ‘encephalitis’, ‘meningitis’ or ‘meningoencephalitis’.

CSF samples were included in the ‘infarction’ group if the sample was derived from a patient who was at the time diagnosed with current ‘cerebral infarction’ of either hemorrhagic or ischemic etiology.

CSF samples were included in the ‘healthy’ group if the sample was derived from a patient who at the time showed no signs or findings of CNS-related diseases or was classified as ‘healthy’.

## Results

### All four SPs are produced by the human central nervous system

#### RT-PCR

**Figure 1:**
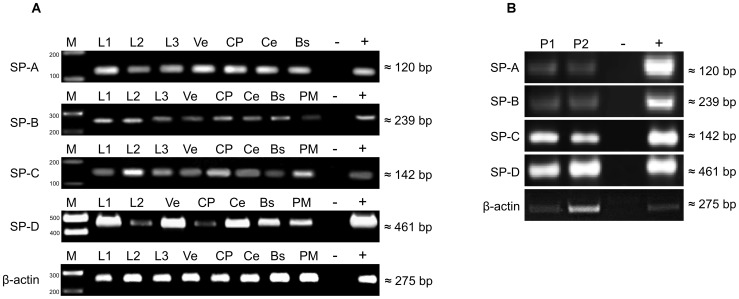
RT-PCR analysis of human brain. Detection of SP-A, SP-B, SP-C and SP-D in **A**) tissue specimens of brainstem (BS), cerebellum (Ce), choroid plexus (CP), subventricular cortex (Ve), pia mater (PM) and cerebrospinal fluid (L1, L2, L3) and **B**) in pineal gland (P1, P2). DEPC-H2O served as negative control (−), RNA-extract from lung tissue was used as positive control (+), molecular size was estimated using a molecular marker (M).

Since examining m-RNA expression from tissues, donated post mortem and snap frozen 9–24 hours after death may have lead to results difficult to interpret, it was decided not to aim for quantitative PCR analysis. Standard RT-PCR was conducted to prove the expression of m-RNA corresponding to the surfactant proteins A, B, C and D only to provide evidence that surfactant proteins are inherent proteins of the CNS. Real time PCR analysis of human CNS tissue, obtained from the living individual or adequate ex vivo testing would provide a much better insight into the distribution and regulation of the surfactant protein system of the human CNS. The fact that unaltered, fresh human tissue specimens, especially samples of the CNS are almost impossible to obtain (except for encephalopunctures performed for further diagnosis of brain pathologies) and the lack of controllability of the post mortal interval were the causes for not conducting real time PCR instead of standard RT-PCR. Specific cDNA amplification products (SP-A: 120 bp, SP-B: 239 bp, SP-C: 142 bp and SP-D: 461 bp) were detected in tissue samples (n = 7 for each tissue) of brainstem (BS), cerebellum (Ce), chorioid plexus (CP), subventricular cortex (Ve), pia mater (PM) cerebrospinal fluid (L1, L2, L3) see Fig. 1A and in pineal gland samples (P1, P2) see Fig. 1B. β-actin RT-PCR was used as a control PCR, see Fig. (1A), Fig. (1B). The control PCR was positive and revealed an equal amount for each tissue in Fig. (1A). In Figure (1B) the expression of m-RNA for beta actin varied between the investigated samples. The specimens investigated are tissue samples from lungs and pineal glands, both organs consist of areas with a high amount of extracellular matrix and distinct areas with higher cellularity. The samples we used for molecular analysis were not investigated microscopically prior to protein extraction or RNA extraction. Therefore we assume that the specimens we examined using RT-PCR probably had a different histological composition, which results in different expression of m-RNA due to a varying relation of extracellular matrix and cellularity. Base pair values were equivalent to the expected DNA products in comparison to gene bank data using the designed primers.

#### Western blot

**Figure 2:**
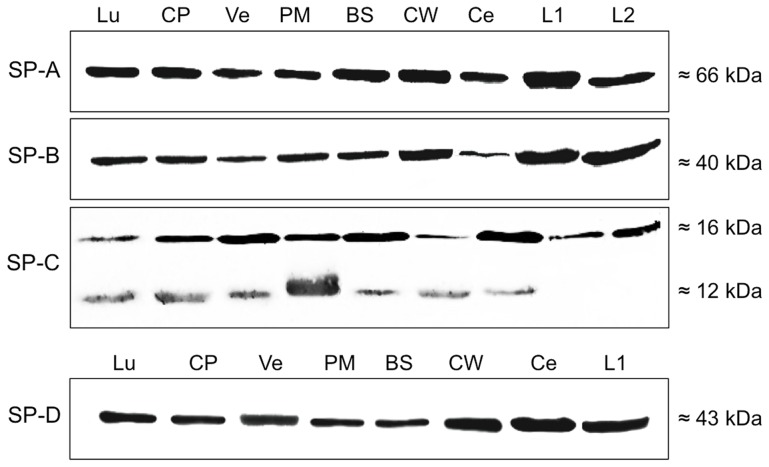
Western blot analysis of surfactant proteins. Detection of SP-A, SP-B, SP-C and SP-D after SDS gel electrophoresis in protein isolates of brainstem (BS), cerebellum (Ce), choroid plexus (CP) the circle of Willis (CW), subventricular cortex (Ve), leptomeninx (PM) and cerebrospinal fluid (L1, L2), Lung (Lu) served as positive control.

Protein isolates (n = 7 for each tissue) of brainstem (BS), cerebellum (Ce), choroid plexus (CP) the circle of Willis (CW), subventricular cortex (Ve), pia mater (PM) and cerebrospinal fluid (L1, L2) were tested for SP-A, SP-B, SP-C and SP-D using specific antibodies to each of the SPs, lung (Lu) served as positive control. Distinct bands were detected for SP-A (pre-mature form, 66 kDa), SP-B (40 kDa), SP-C (16 kDa) and SP-D (43 kDa) in each of the samples, see Fig. (2). In the case of SP-C, a distinct band occurred at 12 kDa in all samples except of cerebrospinal fluid (L1, L2). Protein isolate from lung tissue was treated and incubated under the same conditions and showed distinct bands at the same molecular weight as the CNS-derived samples.

### The hydrophobic surfactant proteins SP-B and SP-C are produced by cells, which contact the cerebrospinal fluid, whereas the distribution of the collectin type SPs aberrates from this pattern

#### Immunohistochemistry

Paraffin-embedded 6 µm sections of choroid plexus, cerebral cortex, pineal gland and rhomboid fossa/brainstem were investigated for presence of SP-A, SP-B, SP-C and SP-D. Lung tissue was used as positive control and revealed positive immunoreactivity for all 4 SPs) not shown here, but already shown in previous work [27], [28]. Negative control (secondary antibody only) was performed and showed no reactivity.

**Figure 3:**
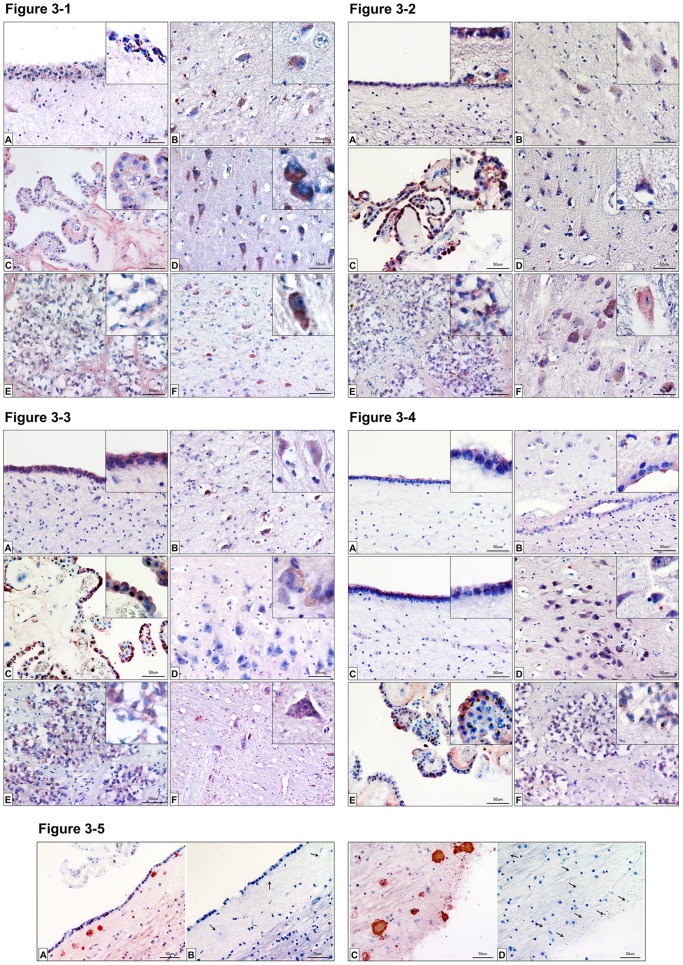
Immunhistochemistry of surfactant proteins A, B, C and D. Figure 3-1: Immunohistochemistry of SP-A: Detection of surfactant protein A in the CNS by means of immunohistochemistry. Sections from the ventricles [A], the hilum region of the hippocampus [B], choroid plexus [C], the dentate gyrus [D], pineal gland [E] and medulla oblongata [F] were analyzed. Red staining indicates SP-A occurrence. Insets in the figures show magnifications for the respective tissue. Control sections (secondary antibody only) were negative (unstained) for each tissue. Scale bars: 50 µm. Figure 3-2: Immunohistochemistry of SP-B: Detection of surfactant protein B in the CNS by means of immunohistochemistry. Sections from the ventricles [A], the hilum region of the hippocampus [B], choroid plexus [C], the dentate gyrus [D], pineal gland [E] and medulla oblongata [F] were analyzed. Red staining indicates SP-B occurrence. Insets in the figures show magnifications for the respective tissue. Control sections (secondary antibody only) were negative (unstained) for each tissue. Scale bars: 50 µm. Figure 3-3: Immunohistochemistry of SP-C: Detection of surfactant protein C in the CNS by means of immunohistochemistry. Sections from the ventricles [A], the hilum region of the hippocampus [B], choroid plexus [C] the dentate gyrus [D], pineal gland [E] and medulla oblongata [F] were analyzed. Red staining indicates SP-C occurrence. Insets in the figures show magnifications for the respective tissue. Control sections (secondary antibody only) were negative (unstained) for each tissue. Scale bars: 50 µm. Figure 3-4: Immunohistochemistry of SP-D: Detection of surfactant protein D in the CNS by means of immunohistochemistry. Sections from the ventricles [A, C], the hilum region of the hippocampus [B] and near the dentate gyrus [D], choroid plexus [E], and pineal gland [F] were analyzed. Red staining indicates SP-D occurrence. Inserts in the figures show magnifications for the respective tissue. Control sections (secondary antibody only) were negative (unstained) for each tissue. Scale bars: 50 µm. Figure 3-5: Immunohistochemistry of SP-C plaques: Detection of surfactant protein C in the CNS by means of immunohistochemistry. Sections from fossa rhomboidea stained [A–C], and unstained [C–D] for SP-C were analyzed. Red staining indicates SP-C occurrence. Insets in the figures show magnifications for the respective tissue. Control sections (secondary antibody only) were negative (unstained) for each tissue. Arrows indicate the possible plaque sites, neuromelanin granules and possibly degenerated neurons. Scale bars: 50 µm.

***SP-A*** is located both diffuse in the cytoplasm and in more intensively stained granular structures in the ependymal cell layer in the ventricular region around the hippocampus. Some IR for SP-A is also present in the subependymal region (Fig. 3, 3-1A). Similar granular location and some staining in neuronal somatais evident in the hilus region of the hippocampal formation (Fig. 3, 3-1B). Strong immunoreactivity for SP-A is expressed by the apical part of the outer layer of the choroid plexus (Fig. 3, 3-1C) and somata of pyramid cells of the dentate gyrus (Fig. 3, 3-1D). The strong somatic staining in these pyramid cells is seen at higher magnification in the insert of 3-1D. In the pineal organ SP-A-IR is present in some pinealocytes and the interlobular structures around the large groups of pinealocytes (Fig. 3, 3-1E). In a horizontal section perpendicular to the brain stem axis of Meynert in the region of the lateral and medial vestibular nucleus and the nucleus of the abducens nerve, some cells, presumably neurons, display somatic immunoreactivity for SP-A (Fig. 3, 3-1F).

***SP-B*** is present in the apical part the ependymal cell layer in the ventricular region around the hippocampus. The basal region is less strongly stained compared to the apical but also immunoreactive. IR for SP-B is also found in the subependymal region in both granular and fibrose structures (Fig. 3, 3-2A). Diffuse staining is evident in neuronal somata of the hippocampal hilus region (Fig. 3, 3-2B). Strong immunoreactivity for SP-B is expressed by the apical and basal part of the outer layer of the choroid plexus (Fig. 3, 3-2C) and somata of pyramid cells of the dentate gyrus (Fig. 3, 3-2D). In the pineal organ SP-B-IR is present in pinealocytes in both diffuse and granular form (Fig. 3, 3-2E). In a horizontal section perpendicular to the brain stem axis of Meynert in the region of the lateral and medial vestibular nucleus and the nucleus of the abducens nerve magnocellular neurons exert somatic immunoreactivity for SP-B (Fig. 3, 3-2F).

***SP-C*** is present in the apical and basal part the ependymal cell layer in the ventricular region around the hippocampus (Fig. 3, 3-3A). Cytoplasmic staining is found in neuronal somata of the hippocampal hilus region (Fig. 3, 3-3B). Strong immunoreactivity for SP-C is expressed by the apical and basal part of the outer layer of the choroid plexus (Fig. 3, 3-3C). Only faint signals are seen in the somata of dentate gyrus pyramid cells (Fig. 3, 3-3D). In the pineal organ SP-C-IR is present in pinealocytes in both diffuse and granular form (Fig. 3, 3-3E). In a horizontal section perpendicular to the brain stem axis of Meynert around the nucleus of the abducens nerve in close proximity to abducens nerve fibers some magnocellular neurons exert somatic immunoreactivity for SP-C (Fig. 3, 3-3F).

Strong immunoreactivity for SP-C is found, among others, in the region of the fossa rhomboidea (Fig. 3, 3–5A, C). Protein plaques, neuromelanin granules and degenerated cellular structures were positive for SP-C.

***SP-D*** is present in the apical part the ependymal cell layer in the ventricular region around the hippocampus (Fig. 3, 3–4A/C). Endothelial staining of large vessels is found in the hippocampal hilus region (Fig. 3, 3-4B). Strong immunoreactivity for SP-D is present in the somata of dentate gyrus small pyramid cells (Fig. 3, 3-4D). Immunoreactivity for SP-D is strongly expressed by the apical part of the outer layer of the choroid plexus, the basal parts is less strongly stained (Fig. 3, 3-4E). In the pineal organ SP-D-IR is present in pinealocytes in both diffuse and granular form (Fig. 3, 3-4F).

**Figure 4:**
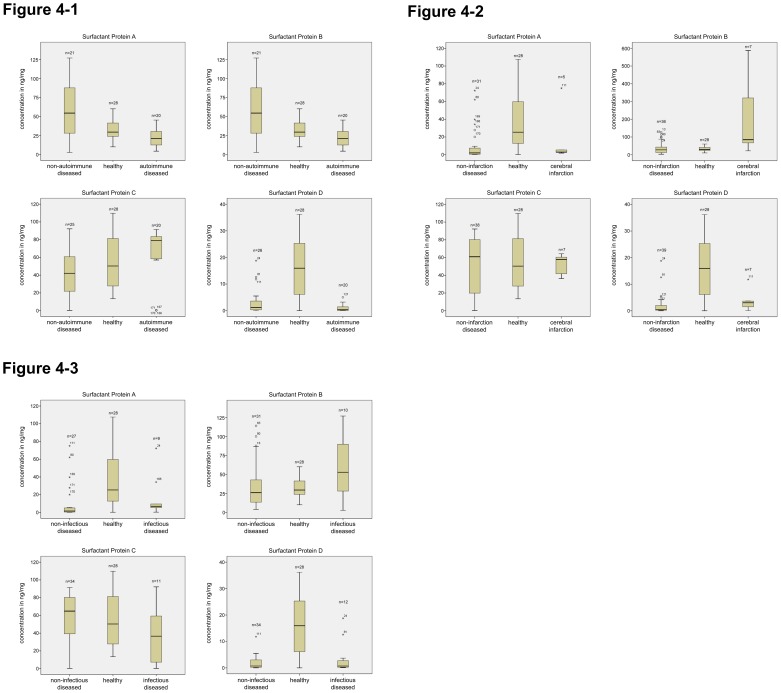
ELISA Quantification of surfactant proteins A, B, C and D in autoimmune diseases, cerebral infarction and infections of the CNS. Figure 4-1: ELISA quantification of surfactant proteins A, B, C and D from autoimmune diseased vs. non-autoimmune diseased vs. healthy. ELISA derived from the following samples: cerebrospinal fluid from different patients, with non-autoimmune diseased, with autoimmune diseased and healthy. The protein concentration is expressed in ng/mg. (P≤0.05). Figure 4-2: ELISA quantification of surfactant proteins A, B, C and D from cerebral infarction vs. non-infarction diseased vs. healthy. ELISA derived from the following samples: cerebrospinal fluid from different patients, with non-infarction diseased, with cerebral infarction and healthy. The protein concentration is expressed in ng/mg. (P≤0.05). Figure 4-3: ELISA quantification of surfactant proteins A, B, C and D from cerebral infection vs. non-infection diseased vs. healthy. ELISA derived from the following samples: cerebrospinal fluid from different patients, with non-infectious diseased, with infectious diseased and healthy. The protein concentration is expressed in ng/mg. (P≤0.05).

### Quantification of surfactant proteins A, B, C and D in the CSF – ELISA and subsequent statistical analysis

#### Autoimmune diseased vs. non autoimmune diseased vs. healthy (Figure 4, 4-1, Table 4): Surfactant protein A

A total of 64 samples were analyzed. 18 (11 female, 7 male) CSF samples derived from patients suffering from a CNS disease with autoimmune etiology were compared to 18 (5 female, 13 male) samples derived from patients suffering from a CNS condition with non-autoimmune etiology and 28 (10 female, 18 male) samples derived from healthy subjects.

**Table 4:** Differences of SP concentration in CSF: diseased groups compared to the healthy control group. Legend: ↑↑ increased, p<0.05, ↑ increased, p>0.05 ↓↓ decreased, p<0.05 ↓ decreased, p>0.05 =  no difference.

	Autoimmune vs. healthy	cerebral infarction vs. healthy	cerebral infection vs. healthy
**SP-A**	**↑↑**	**↓**	**↓↓**
**SP-B**	**↓↓**	**↑↑**	**↑**
**SP-C**	**↑**	** = **	**↓**
**SP-D**	**↓↓**	**↓↓**	**↓↓**

The level of SP-A concentration in CSF from patients with autoimmune CNS disease was statistically significantly decreased (p = 0.01) compared to the level of SP-A concentration in the healthy group.

#### Surfactant protein B

A total of 69 samples were analyzed. 20 (11 female, 9 male) CSF samples derived from patients suffering from a CNS disease with autoimmune etiology were compared to 21 (5 female, 16 male) samples derived from patients suffering from a CNS condition with non-autoimmune etiology and 28 (10 female, 18 male) samples derived from healthy subjects.

Statistical analysis revealed that the level of SP-B in CSF from patients with an autoimmune CNS disease was significantly decreased (p = 0.015) compared to the level of SP-B in the healthy group.

#### Surfactant Protein C

A total of 73 samples were analyzed. 20 (11 female, 9 male) CSF samples derived from patients suffering from a CNS disease with autoimmune etiology were compared to 25 (7 female, 18 male) samples derived from patients suffering from a CNS condition with non-autoimmune etiology and 28 (10 female, 18 male) samples derived from healthy subjects.

The level of SP-C in CSF from patients with an autoimmune CNS disease was comparable to the level of SP-C in the healthy group (p = 0.415).

#### Surfactant Protein D

A total of 74 samples were analyzed. 20 (11 female, 9 male) CSF samples derived from patients suffering from a CNS disease with autoimmune etiology were compared to 26 samples (7 female, 19 male) derived from patients suffering from a CNS condition with non-autoimmune etiology and 28 (10 female, 18 male) samples derived from healthy subjects.

The level of SP-D in CSF from patients with an autoimmune CNS disease was decreased in a statistically significant manner (p = 0.01) compared to the level of SP-D in the healthy group.

#### Cerebral infarction vs. non-infarction diseased vs. healthy (Figure 4, 4-2, Table 4): Surfactant Protein A

A total of 64 samples were analyzed. 5 (2 female, 3 male) CSF samples derived from patients who had suffered a cerebral infarction were compared to 31 (16 female, 15 male) samples derived from patients suffering from a CNS condition not related to cerebral infarction and 28 (10 female, 18 male) samples derived from healthy subjects.

The level of SP-A in CSF from patients with cerebral infarction was slightly decreased, but did not reach statistical significance (p = 0.083) compared to the level of SP-A in the healthy group.

#### Surfactant Protein B

A total of 71 samples were analyzed. 7 (2 female, 5 male) CSF samples derived from patients who had suffered a cerebral infarction were compared to 36 (19 female, 17 male) samples derived from patients suffering from a CNS condition not related to cerebral infarction and 28 (10 female, 18 male) samples derived from healthy subjects. The level of SP-B in CSF from patients with cerebral infarction was statistically significantly increased (p = 0.002) in comparison to the level of SP-B in the healthy group.

#### Surfactant Protein C

A total of 73 samples were analyzed. 7 (2 female, 5 male) CSF samples derived from patients who had suffered a cerebral infarction were compared to 38 (19 female, 19 male) samples derived from patients suffering from a CNS condition not related to cerebral infarction and 28 (10 female, 18 male) samples derived from healthy subjects. The level of SP-C in CSF from patients with cerebral infarction showed no difference (p = 0.967) compared to the level of SP-C in the healthy group.

#### Surfactant Protein D

A total of 73 samples were analyzed. 7 (2 female, 5 male) CSF samples derived from patients who had suffered a cerebral infarction were compared to 39 (19 female, 20 male) samples derived from patients suffering from a CNS condition not related to cerebral infarction and 28 (10 female, 18 male) samples derived from healthy subjects. The level of SP-D in CSF from patients with cerebral infarction was statistically significantly decreased compared to the level of SP-D in the healthy group (p = 0.012).

#### Cerebral infection vs. non-infection diseased vs. healthy (Figure 4, 4-3, Table 4): Surfactant Protein A

A total of 64 samples were analyzed. 9 (3 female, 6 male) CSF samples derived from patients diagnosed at the time with an infectious disease of the CNS were compared to 27 (13 female, 14 male) samples derived from patients suffering from a different disorder of the CNS than infection and 28 (10 female, 18 male) samples derived from healthy subjects. The level of SP-A in CSF from patients with a CNS infection was statistically significantly decreased (p = 0.044) compared to the level of SP-A in the healthy group.

#### Surfactant Protein B

A total of 69 samples were analyzed. 10 (3 female, 7 male) CSF samples derived from patients diagnosed at the time with an infectious disease of the CNS were compared to 31 (13 female, 17 male) samples derived from patients suffering from a different disorder of the CNS than infection and 28 (10 female, 18 male) samples derived from healthy subjects.

The level of SP-B in CSF from patients with a CNS infection was slightly increased, but did not reach statistical significance compared to the level of SP-A in the healthy group (p = 0.083).

#### Surfactant Protein C

A total of 73 samples were analyzed. 11 (3 female, 8 male) CSF samples derived from patients diagnosed at the time with an infectious disease of the CNS were compared to 34 (14 female, 20 male) samples derived from patients suffering from a different disorder of the CNS than infection and 28 (10 female, 18 male) samples derived from healthy subjects. The level of SP-C in CSF from patients with a CNS infection was slightly decreased, but did not reach statistical significance (p = 0.072) compared to the level of SP-C in the healthy group.

#### Surfactant Protein D

A total of 74 samples were analyzed. 12 (4 female, 8 male) CSF samples derived from patients diagnosed at the time with an infectious disease of the CNS were compared to 34 (14 female, 20 male) samples derived from patients suffering from a different disorder of the CNS than infection and 28 (10 female, 18 male) samples derived from healthy subjects. The level of SP-D in CSF from patients with a CNS infection was statistically significantly decreased (p = 0.001) in comparison to the level of SP-D in the healthy group.

### Quantification of surfactant proteins A, B, C and D in human brain tissue – ELISA

ELISA was performed with protein extracts from subventricular cortex, brainstem, cerebellum and choroid plexus from body donors with comparable postmortal intervals. The surfactant protein concentration in brain tissue (except the choroid plexus) was measured to be approximately twenty five percent of the surfactant protein concentration in the lungs. The surfactant protein concentrations of the cerebellum, the subventricular cortex and the brainstem were in equal range, whereas the tissue concentration of all SPs in the choroid plexus was approximately twofold higher compared to the concentration in brainstem, cerebellum and subventricular cortex.

## Discussion

By use of RT-PCR we were able to verify the intrinsic production of messenger RNA for all investigated SPs by the central nervous system. The fact that we found mRNA for the SPs in the cerebrospinal fluid is unexpected. We hypothesize that the mRNA we found is a result of the cell damage due to the trauma of the lumbar puncture, which always leads to limited destruction of cells (especially meningeal cells) in the area of the puncture. The cell damage results in the liberation of intracellular components like proteins and mRNA. We address this as the reason for the detection of SP-specific mRNA in the CSF. The results of Western blot and immunohistochemical analysis confirm our findings on protein level. Based on proof of both the production of SPs in the human CNS and the expression of SP-specific mRNA, our study reveals the existence of surfactant proteins as inherent proteins of the CNS.

In the case of SP-C, the antibody used detected premature forms of the proteins at 12 kDa only in the non-CSF tissue. This issue is well known and a consequence of an intense posttranslational processing of the SPs. We therefore conclude that mature SP-C does not play an important role in host defense or amelioration of inflammation in CNS diseases.

The collectin-type SPs A and D showed immunoreactivity in the tissue surrounding the microvasculature of the brain parenchyma, the capillaries of choroid plexus stroma and the small vessels at the base of the pineal gland. Both proteins were detected in the vascular layers (with the exception of the tunica intima) as well as in the adjacent brain tissue. Their volumetric abundance between the blood milieu of the cerebral vessels and the brain tissue, especially at the level of the glia limitans (formed by astrocytic endfeet) and the perivascular Virchow-Robin space, resembling the microanatomical correlate of the blood-brain barrier, indicates a participation in this convolute of CNS-protective mechanisms. In lung, SP-A and SP-D are known to be antimicrobially active proteins of the alveolar surface. They are localized in the surfactant layer lining the alveolar surface as well as within the apical portion of the type II alveolar epithelial cells. One of their tasks is to bind microbial invaders like Pseudomonas aeruginosa or Staphylococcus aureus, to mediate their elimination via direct bactericidal effects or indirectly by means of opsonization and subsequent phagocytosis by macrophages [31]. Considering our findings and the well-known functions of SP-A and SP-D, we hypothesize that SP-A and SP-D are pathogen-binding proteins that prevent the invasion of the CNS by microbial invaders circulating with the blood stream. The collectin-type SPs may directly kill extravasating bacteria on their way into the brain tissue. They could furthermore facilitate the elimination of invading microbes by opsonization via their carbohydrate recognition domain and following binding to surveilling perivascular microglia. Finally, in case of a CNS infection, they may trigger the shift from an anti-inflammatory to a pro-inflammatory milieu in the CNS by binding to resting microglia, with subsequent activation and production of pro-inflammatory mediators [5]. Another group of antimicrobial peptides, the human β-defensins 1 and 2, is expressed by cells at both sites, the „air lung barrier“ [32] and the blood brain barrier [33]. Human β-defensin 1 and 2 are produced by epithelial cells at different sites of the body, e.g. in the intestinal mucosa, the female reproductive tract and the lungs. They enhance the clearance of microbial pathogens in the human body by directly killing the pathogens (perforation of bacterial walls) and mediate the uptake of antigens by binding to specific receptors on phagocytotic cells [34]. Another human β-defensin, DEFB123, has been shown to bind bacterial lipopolysaccharides and prevent the lethal consequences of sepsis in a mouse model [35]. The similar functions and the resemblance in the distribution patterns of the two systems – the collectin-type SPs and the human β-defensins, especially at anatomical-physiological barriers in different tissues, underlines the possible importance of SP-A and SP-D as players in innate immunity at the BBB.

The lipophilic SPs show immunoreactivity in the epithelial layer of the choroid plexus, in the ependymal cells lining the four ventricles of the brain and in the ependyma of the spinal channel. The choroid plexus and the ependymal cells of the ventricles are the main production site of the cerebrospinal fluid [36], [37]. The production, flow and resorption of CSF have to be closely regulated. A decreased production, an impaired flow or an insufficient resorption of CSF rapidly lead to altered intracranial pressure, thus resulting in severely neurological conditions such as spontaneous intracranial hypotension, idiopathic intracranial hypertension and normal pressure hydrocephalus [38]. Neither of the underlying pathomechanisms for these conditions is well understood.

The function and physiological importance of SP-B and SP-C has been studied in the lungs, the auditive tube, the tear fluid and the lacrimal system among other sites [18], [28], [39], [40]. Both proteins are crucial for the formation and stability of surface-active monolayers and contribute to lowering the surface tension, thus preventing the collapse of the alveoli at the end of expiration [41]. They furthermore regulate the permeability of surfactant phospholipid membranes [20]. Inherited defects of SP-B and SP-C lead to respiratory failure and interstitial lung disease in early childhood [42]. Considering their ability to decrease surface tension, modulate the permeability of biomembranes and lower the viscosity of phospholipid layers [43], we assume that SP-B and SP-C are essential for the continuous secretion of CSF, the maintenance of its rheological properties and correct drainage into the arachnoid villi of the cerebral venous sinuses. We furthermore hypothesize that disturbances of the metabolism of SP-B and SP-C may be of pathogenic importance for conditions like spontaneous intracranial hypotension, idiopathic intracranial hypertension and normal pressure hydrocephalus. In our analysis of immunohistochemistry of surfactant protein C, we found plaque-like structures in many areas of the brain. The staining of SP-C is specific and we propose that SP-C may play an interesting role in the development of plaques like those found in the brains of Alzheimer's patients. It is known that SP-C forms amyloid structures (“plaques”) as a result of misfolding of the proteins [44], [45]. Comparing the plaque images from other publications with the SP-C immunoreactivity described here, we conclude that what we have here is the first positive evidence for the presence of SP-C in human brain plaques [46], [47]. In this context it is assumed that amyloid structures exist due to failure of the clearance system via the glymphatic pathway. The failure of this clearance system contributes to amyloid plaque deposition and Alzheimer's disease progression [48].

The choroid plexus and the ventricular ependyma also produce phospholipid transfer protein, another protein participating in the formation of the surfactant layer in the lungs [49], [50]. After this initial description of a protein at the blood-cerebrospinal fluid barrier, which plays an important role for the decrease of surface tension within the alveoli, we now report the existence of a second functionally related protein system at this particular location in the CNS – the lipophilic SPs. We understand the abundance of both systems at the blood-CSF barrier as a strong indicator for the hypothesis that surface-active proteins of the lung are also utilized to maintain the rheological properties of cerebrospinal fluid in order to perpetuate a physiological CSF flow.

The concentration of the collectin-type SPs in CSF samples from patients with autoimmune diseases, infectious conditions and CNS infarctions was significantly lower compared to samples derived from healthy subjects. It thus appears that both proteins are involved in inflammatory processes in the CNS. Considering the known functions of these proteins, we assume their contribution in the local immune response. SP-A and SP-D are involved in the clearance of pathogens and apoptotic polymorph nuclear neutrophils [51], [52].

Polymorph nuclear neutrophils are among the first wave of innate immune cells at inflammation sites, they release a whole arsenal of anti-microbial molecules and their survival is closely regulated to prevent tissue damage caused by excessive neutrophil activity [53]. The collectin-type SPs may be involved in the elimination of both exhausted neutrophils and microbial invaders in the CNS. Similar to the situation in lung they may bind to the pathogen or the apoptotic cell and subsequently mediate the phagocytosis via CD91 and calreticulin [54]. It has been shown that SP-A and SP-D are effective opsonins for apoptotic cells. Like mannose-binding lectin (MBL) and the complement factor C1q, SP-A and SP-D label cells that have undergone programmed cell death for macrophages to facilitate their phagocytosis [55], [56].

As a consequence of this, the collectin-type SPs may be removed from the CSF of patients with inflammatory CNS conditions like infarctions, infections or autoimmune diseases during the phagocytosis of (SP-) opsonized targets.

The concentration of SP-B in CSF samples from patients with autoimmune CNS conditions was significantly lower compared to samples from healthy subjects. By contrast, the SP-B concentration in CSF samples from patients with infections and infarctions of the CNS was significantly higher than the concentration in samples from the healthy group.

The phenomenon of high SP-B concentration in the CSF of patients with inflammation of non-autoimmune etiology and low SP-B concentration in the CSF of patients with autoimmune etiology probably has different underlying mechanisms.

SP-B possesses anti-inflammatory properties and has the potential to reverse inflammation in the lungs [21]. It also inhibits endotoxin-induced lung inflammation [57]. The concentration of SP-B in amniotic fluid of patients with intra-amniotic infection is significantly increased compared to that of healthy pregnant women [58].

Consistent with these findings, we propose that SP-B is upregulated during exogenously induced inflammation in order to limit and counterbalance the detrimental effects caused by the host's immune system. Specifically, we hypothesize that SP-B is a protein of the CSF, constitutively produced by the choroid plexus to facilitate CSF flow with a reactively increased production rate in case of pathogen-induced or ischemically triggered, acute neuroinflammation.

The decreased concentration of SP-B in CSF samples from patients with autoimmune CNS conditions may be the consequence of a continuous depletion of SP-B due to the chronicity of autoimmune diseases. Autoimmune CNS conditions like multiple sclerosis are coupled with permanent inflammation, influx of immune cells and cell death in the CNS [59]. The persistent chronic inflammation may lead to a high consumption of SP-B, resulting in a functional insufficiency of the choroid plexus and the ependymal cells to produce SP-B, finally leading to a decreased concentration of SP-B in the CSF. The involvement of SP-B in autoimmune conditions has not been investigated as yet to our knowledge. A thorough review of the literature on this issue revealed no evidence of studies focusing on lipophilic SPs in autoimmune diseases.

We were able to detect SP-C in all of the investigated CSF samples, but there was no difference in the concentration of SP-C between the different groups. We therefore conclude that mature SP-C does not play an important role in host defense or amelioration of inflammation in CNS diseases. Western blot analysis did not reveal the existence of the 12 kDa premature form of SP-C in the CSF. It is therefore conceivable that this premature form may be regulated differently in the investigated groups of CNS conditions, but the ELISA system we used may also not be capable of detecting the 12 kDa form.

### Conclusion

The ‘pulmonary’ surfactant proteins are also inherent proteins of the human CNS. The collectin-like SPs – SP-A and SP-D show a similar distribution pattern in the CNS, which varies from the distribution pattern of the hydrophobic SPs SP-B and SP-C. Their expression at different locations in the CNS indicates participation of the SPs in processes like host defense and maintenance of CSF flow, but the dimension of their importance and their exact function must be investigated in subsequent studies.

The concentration of surfactant proteins in the CSF of healthy subjects deviates from patients with CNS conditions of autoimmune, ischemic and infectious etiology. This also speaks for the importance of the SPs in processes such as fine-tuning of inflammation and host defense. It is still not clear which cells are exactly responsible for production of the collectin-type SPs within the CNS. This remains to be investigated prior to more functional studies.
