# Correction: The Detection of Surfactant Proteins A, B, C and D in the Human Brain and Their Regulation in Cerebral Infarction, Autoimmune Conditions and Infections of the CNS

**DOI:** 10.1371/annotation/920eb90c-4f7f-4468-ae9c-1198b7b952fc

**Published:** 2013-11-12

**Authors:** Stefan Schob, Martin Schicht, Saadettin Sel, Dankwart Stiller, Alexander S. Kekulé, Friedrich Paulsen, Erik Maronde, Lars Bräuer

The first two authors, Stefan Schob and Martin Schicht, should be noted as contributing equally to this work. The last two authors, Erik Maronde and Lars Bräuer, should be noted as contributing equally to this work.

The fifth author's name was spelled incorrectly. The correct name is Alexander S. Kekulé.

The correct citation is: Schob S, Schicht M, Sel S, Stiller D, Kekulé AS, et al. (2013) The Detection of Surfactant Proteins A, B, C and D in the Human Brain and Their Regulation in Cerebral Infarction, Autoimmune Conditions and Infections of the CNS. PLoS ONE 8(9): e74412. doi:10.1371/journal.pone.0074412

